# Prevalence of Hyposalivation in Patients with Systemic Lupus Erythematosus in a Brazilian Subpopulation

**DOI:** 10.1155/2015/730285

**Published:** 2015-01-11

**Authors:** Cristhiane Almeida Leite, Marcial Francis Galera, Mariano Martínez Espinosa, Paulo Ricardo Teles de Lima, Vander Fernandes, Álvaro Henrique Borges, Eliane Pedra Dias

**Affiliations:** ^1^Department of Oral Pathology, University of Cuiabá, Avenida Manoel José de Arruda 3.100 Jardim Europa, 78065-900 Cuiabá, MT, Brazil; ^2^Department of Pathology, Federal Fluminense University, Rua Marquês do Paraná 303, No. 4 Andar do Prédio Principal, Sala 1, 24.033-900 Niterói, RJ, Brazil; ^3^Department of Pediatrics, School of Medicine, Federal University of Mato Grosso, Avenida Fernando Corrêa da Costa 2367, Boa Esperança, 78060-900 Cuiabá, MT, Brazil; ^4^Department of Statistics, Institute of Exact Sciences, Federal University of Mato Grosso, Avenida Fernando Corrêa da Costa 2367, Boa Esperança, 78060-900 Cuiabá, MT, Brazil; ^5^School of Dentistry, University of Cuiabá, Brazil Avenida Manoel José de Arruda 3.100, Jardim Europa, 78065-900 Cuiabá, MT, Brazil; ^6^Department of Rheumatology, University of Cuiabá, Avenida Manoel José de Arruda 3.100, Jardim Europa, 78065-900 Cuiabá, MT, Brazil; ^7^Faculty of Dentistry, University of Cuiabá, Avenida Manoel José de Arruda 3.100, Jardim Europa, 78065-900 Cuiabá, MT, Brazil

## Abstract

*Background.* Systemic lupus erythematosus (SLE) is a chronic inflammatory, multisystem, and autoimmune disease. *Objective.* The aim of this study was to describe the prevalence of hyposalivation in SLE patients and evaluate factors associated. *Methods.* This is a cross-sectional study developed at the Cuiaba University General Hospital (UNIC-HGU), Mato Grosso, Brazil. The study population consisted of female SLE patients treated at this hospital from 06/2010 to 12/2012. Unstimulated salivary flow rates (SFRs) were measured. Descriptive and inferential analyses were performed in all cases using a significance level *P* < 0.05. *Results.* The results showed that 79% of patients with systemic lupus erythematosus suffered from hyposalivation and that the disease activity and age in years were the factors that resulted in statistically significant differences. *Conclusion.* The activity of the disease, age >27 years, and the drugs used were factors associated with hyposalivation, resulting in a statistically significant decrease in saliva production.

## 1. Introduction

Systemic lupus erythematosus (SLE) is a systemic autoimmune and inflammatory disease that affects many organs and systems through formation and deposition of autoantibodies and immune complexes leading to severe tissue and organ damage [1, 2]. It is characterized by hyperreactivity of T and B cells and a failure to eliminate apoptotic bodies [2–4]. Patients with SLE may present with several oral manifestations, the prevalence varies from 20 to 80%, and usually more than one injury is present [5–13].

The terms salivary hypofunction or hyposalivation and xerostomia are often incorrectly used interchangeably. Hyposalivation refers to a diminished salivary flow, whereas xerostomia refers to a subjective experience of mouth dryness. This is further complicated by the fact that some patients with hyposalivation are not xerostomic and, conversely, those with xerostomia may have normal salivary flow rates. However, xerostomia is a common and primary symptom associated with salivary gland hypofunction. Usually when salivary secretion has decreased to half its normal values an individual will begin to experience xerostomia [14].

More than 75% of patients with SLE suffer from oral complaints like dryness (xerostomia) and soreness [9]. Systemic lupus erythematosus has also been associated with a decrease in salivary flow, resulting in xerostomia and hyposalivation has already been described in these patients [5–11, 13, 15–17]. This dysfunction in the salivary glands and the detection of salivary changes present in SLE patients can reflect a distinct and specific multisystem presentation [16, 17]. Ben-Aryeh et al. 1993 [15] studied a group of SLE patients with no other systemic diseases and none of the patients complained of xerostomia. Yet, those patients had significantly lower salivary flow rates than controls. In other studies, patients with SLE experienced some degree of xerostomia [17] and had significantly lower SWS compared with healthy controls [18].

The complexity of the molecular composition of saliva has shown its importance related to the maintenance of oral and systemic integrity, and it is critical for the first line of oral defense. Functions of saliva include tissue repair (presence of epidermal growth factor (EGF) promotes healing of the oral, oropharynx, and gastric mucosa), protection (lubrication of the mouth, oropharynx, and esophagus), tamponage (phosphate, bicarbonate, and proteins maintain unfavorable pH for microorganism colonization, neutralization of acidity), digestion (formation of the food bolus and digestion of starch, proteins, and lipids), gustation (solubilization of molecules and maturation of taste buds), antimicrobial action (presence of antibodies IgA/IgM and IgG, lysozyme and lactoferrin-bacterial antagonism, system of peroxidase/cystatin/mucin, and immunoglobulins-antiviral activity, histatin/chromogranin A, and immunoglobulins-antifungal activity), and maintenance of tooth integrity (maturation of the enamel and remineralization) [9, 19–22]. In addition, patients may experience halitosis, sleep disorders, dysphagia, and difficulty in swallowing and speaking [23, 24].

The salivary flow rate reduction can be caused by several factors, including a dysfunction in the salivary gland, systemic diseases, age, other autoimmune diseases such as Sjögren's syndrome, and several drugs [13, 14, 16, 21, 25–27]. Although some studies investigated the prevalence of hyposalivation in SLE patients [5, 7, 15, 28], none of them employed a scientific approach towards the evaluation of the factors associated with this variable in this group of patients. The aim of this study was to determine the prevalence of hyposalivation in patients with systemic lupus erythematosus and evaluate the factors associated with this variable.

## 2. Materials and Methods

### 2.1. Subjects and Study Design

After approval by the Ethics Committee of the University General Hospital, University of Cuiabá, all patients with SLE in Cuiabá University General Hospital (HGU-UNIC), Mato Grosso, Brazil, from July 2010 to December 2013, were included. The criteria for the diagnosis of SLE were according to the American College of Rheumatology revised classification [29]. A medical history, including information related to current systemic disease, disease activity scores using SLEDAI (*systemic lupus erythematosus disease activity*) [30], and on-going medications, was obtained for all patients. Activity categories have been defined on the basis of SLEDAI scores: no activity (SLEDAI = 0), mild activity (SLEDAI = 1–5), moderate activity (SLEDAI = 6–10), high activity (SLEDAI = 11–19), and very high activity (SLEDAI 20) [31]. Exclusion criteria included the presence of any of the following: previous history of radiation therapy in the head and neck area, poorly uncontrolled diabetes mellitus, chronic thyroid disease, known Sjogren's disease, missing complete data, and not collecting the saliva.

Total salivary flow rates (SFRs) at rest were determined according to the guidelines for collecting unstimulated whole saliva [32]. The participants were asked to collect saliva in their mouth and to split it into a wide test tube for 5 minutes. As a reference, a rate of 0.3 mL/min was considered a normal salivary flow of unstimulated saliva [33]. A value less than 0.3 mL/min in 5 minutes (totaling <1.5 mL/5 minutes) was classified as hyposalivation [33].

Statistical analyses were performed with SPSS and Minitab version 15. Student's *t*-test for two independent samples was used for the inferential analysis to compare the averages of the variables. Multiple linear regression was used in all cases and *P* values below 5% were considered significant.

## 3. Results

Of the 93 patients evaluated (2010–2013), 48 female patients fulfilled all the inclusion criteria and 38 (79.2%) had hyposalivation. The amount of saliva decreased with disease activity; however, this reduction was not statistically significant (*P* = 0.500) based on Student's *t*-test. However, the lowest values were found in patients with higher disease activity (severe and very severe) compared with those who were in remission or had mild or moderate activity, and this average decrease was statistically significant (*P* = 0.004), as demonstrated in Table 1.

When evaluating the relation of drugs used and the amount of saliva, the medications considered as hyposalivation were antihypertensive, anticonvulsant, and diuretic. The amount of saliva decreased with the use of these (*P* = 0.442), especially when using just one of these medications (*P* = 0.089); however, this mean reduction was not statistically significant (*P* = 0.442), as verified by Student's *t*-test.

**Figure 1:**
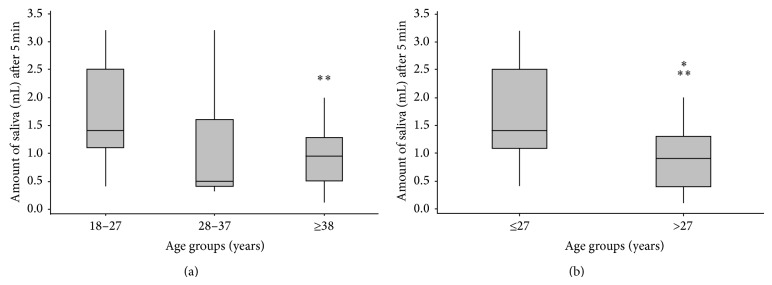
(a) Box plots comparing the amount of saliva by age in years. (b) Box plots comparing the amount of saliva by age in years.

Regarding age in years, three age groups were considered (18–27 years, 28–37 years, and 38 years or older), which showed that the saliva production decreased when we compared the first age group with the other two age groups; however, this decrease was not statistically significant (*P* = 0.059) based on ANOVA test.  Figure 1 shows that age can be analyzed based on two age groups (≤27 years and >27 years old) because the second and third age groups have very similar amounts of saliva (1.01 and 1.09, resp.). These two age groups showed that saliva decreased from less than 27 years to greater than 27 years, and this decrease was statistically significant (*P* = 0.021), as indicated by Student's *t*-test.

Table 1 presents the descriptive statistics, confidence intervals of 95%, and *P* values for the amount of saliva in mL, collected within 5 minutes per category of variables, and shows that the activity level and age group (years) were statistically significant at 5% (*P* = 0.004 and *P* = 0.021, resp.).

## 4. Discussion

This is the first study to evaluate the risk factors for hyposalivation in SLE patients. The prevalence found in this study (79.2%) is higher than in the general population (20%) [20] but within the previously published data [5–11, 13, 15–17]. Few studies have been published in SLE patients [5–11, 13, 15–17] and the differences between these may be due to the differences in the diagnostic criteria of hyposalivation.

Systemic lupus erythematosus is a chronic inflammatory, multisystem disease, and although the involvement of the salivary glands and salivary flow rate is not commonly described in the literature, we observe in clinical practice that patients with SLE frequently complain of xerostomia associated or not with hyposalivation. Authors consider the decrease in salivary flow rate in SLE as a result of secondary Sjogren's syndrome [34–36], but histopathologic features of the minor salivary glands are distinctly different in this syndrome and lupus erythematosus. Alterations in salivary glands of LE patients may be a specific manifestation of the disease (lupus sialadenitis), reflecting its multisystemic presentation, instead of an association of secondary SS [16, 17]. The patients included in this study have not yet been subjected to minor salivary gland biopsy for Sjogren's syndrome, but 18% had positivity for anti-Ro/SSA and 9% for anti-La/SSB.

Hyposalivation has been found among younger adults [37, 38]. Flink et al. 2008 [39] found prevalent hyposalivation in younger adults and this unexpectedly high prevalence in younger age groups indicates that this may be of significance for oral health in these groups of patients. Salivary gland function declined with age and may be related to the number of medications they take on a regular basis, the number of systemic disorders they report, and the length of time for which they consume the drugs [26, 27]. With increasing age, the focus of inflammatory cells increases, and acinar atrophy, ductal dilatation, and variable degrees of fibrosis in the salivary glands are observed [40, 41]. This study shows that age can be analyzed based on two age groups (≤27 years and >27 years old) because the second and third age groups have very similar amounts of saliva (Figure 1). These two age groups showed that saliva decreased from less than 27 years to greater than 27 years, and this decrease was statistically significant (*P* = 0.021). Percival et al. 1994 [42] distributed the patients in four groups (20–39 years, 40–59 years, 60–79 years, and ≥80 years) and a significant decrease in the secretion rates of unstimulated whole saliva in relation to age was also observed in the study population (*P* < 0.001).

During the course of the disease and with the age, SLE patients use several medications [43] that may be interfering with saliva secretion. Medication use is believed to be an important reason for reduction of salivary flow and diuretics, antihypertensives, antihistamines, sedatives, opioid analgesics, tricyclic antidepressives, and major antipsychotics will reduce the flow [26, 27]. Interestingly, we observed that although the use of drugs that decrease saliva, no significant differences were observed in this point. In addition, the worsening on hyposalivation did not depend on the total number of medications ingested.

SLE patients showed a significant reduction in salivary flow rate compared to controls, as well as high concentrations of sodium, calcium, magnesium, and immunoglobulin A (IgA) and IgM antibodies, thus concluding that these changes in salivary composition may represent involvement of salivary glands in these patients [15]. Almost half of the patients with SLE had reduced salivary flow as a result of impaired function of major salivary glands and in some of these cases, however, the patients did not suffer from xerostomia and, even more remarkable, some patients with normal salivary flow did complain of dry mouth [17]. A few studies show that SLE without other autoimmune associated diseases is related to a decreased nonstimulated flow rate of whole saliva [36]. Knowledge of the prevalence of hyposalivation is found in SLE patients but, in this study, we showed that, in addition to disease, patients with higher levels of disease activity had lower amounts of saliva, a fact that reinforces that hyposalivation can directly reflect a dysfunction in the salivary glands of the disease.

In conclusion, we found that the activity of the disease and age >27 years were factors associated with hyposalivation in patients with systemic lupus erythematosus indicating that these factors decrease the amount of saliva in a statistically significant manner.

The prevalence of hyposalivation presented in this study is limited to a relatively homogenous group of patients. However, in the absence of information about hyposalivation in patients with systemic lupus erythematosus in a Brazilian subpopulation, this present study seems to offer the only available information. Future longitudinal studies are needed to learn more about hyposalivation in this group of patients and further studies to confirm this finding.

## Figures and Tables

**Table 1:** Average of saliva collected per variables considered from 48 patients at the HGU-UNIC, Cuiaba, MT, 2014.

Variables	*n*	Averages	Standard derivation	95% CI	*P*
SLE activity					
Yes	38	1,23	0,89	(−0,5; 1,01)	0,500
No	10	1,47	1,01	—	—
Level of SLE activity					
Severe/very severe	10	0,78	0,42	(0,214; 1,04)	0,004
Remission/mild or moderate	38	1,41	0,97	—	—
Ages					
>27	31	1.05	0,85	(0,10; 1,19)	0,021
≤27 years	17	1,69	0,90	—	—
Use of hyposalivation-inducing drugs?					
Yes	10	1,08	0,87	(−0,46; 0,92)	0,442
No	38	1,33	0,93	—	—
Number of hyposalivation-inducing drugs					
0	38	1,33	0,93	—	—
1	4	0,65	0,54	(−0,17; 1,53)	0,089
2	6	1,37	0,98	(−1,14; 0,93)	0,817

95% CI: confidence interval of 95% for the difference between the averages of the categories.
